# A Macrosphelide as the Unexpected Product of a *Pleurotus ostreatus* Strain-Mediated Biotransformation of Halolactones Containing the *gem*-Dimethylcyclohexane Ring. Part 1

**DOI:** 10.3390/molecules21070859

**Published:** 2016-06-30

**Authors:** Katarzyna Wińska, Wanda Mączka, Małgorzata Grabarczyk, Kenji Sugimoto, Yuji Matsuya, Antoni Szumny, Mirosław Anioł

**Affiliations:** 1 Department of Chemistry, Wroclaw University of Environmental and Life Sciences, Norwida 25, 50-375 Wrocław, Poland; wanda_m19@o2.pl (W.M.); magrab@onet.pl (M.G.); antjasz@o2.pl (A.S.); miroslaw.aniol@up.wroc.pl (M.A.); 2 Graduate School of Medicine and Pharmaceutical Sciences, University of Toyama, 2630 Sugitani, 930-0194 Toyama, Japan; ksugimo@pha.u-toyama.ac.jp (K.S.); matsuya@pha.u-toyama.ac.jp (Y.M.)

**Keywords:** macrosphelide, biotransformation, *Pleurotus ostreatus*, hydrolytic dehalogenation

## Abstract

The aim of the study was to obtain new compounds during biotransformation of two halocompounds, the δ-bromo and δ-iodo-γ-bicyclolactones **1** and **2**. Unexpectedly *Pleurotus ostreatus* produced together with the hydroxylactone, 2-hydroxy-4,4-dimethyl-9-oxabicyclo[4.3.0]nonane-8-one (**3**), its own metabolite (3*S*,9*S*,15*S*)-(6*E*,12*E*)-3,9,15-trimethyl-4,10,16-trioxacyclohexa-deca-6,12-diene-1,5,8,11,14-pentaone (**4**). The method presented here, in which this macrosphelide **4** was obtained by biotransformation, has not been previously described in the literature. To the best of our knowledge, this compound has been prepared only by chemical synthesis to date. This is the first report on the possibility of the biosynthesis of this compound by the *Pleurotus ostreatus* strain. The conditions and factors, like temperature, salts, organic solvents, affecting the production of this macrosphelide by *Pleurotus ostreatus* strain were examined. The highest yield of macroshphelide production was noticed for halolactones, as well with iodide, bromide, iron and copper (2+) ions as inductors.

## 1. Introduction

Fungi are remarkable organisms that easily produce a wide range of secondary metabolites with broad spectrum biological activity [1]. Forty-two percent of about 23,000 active compounds from microorganisms are made by fungi and thirty two percent by actinomycete—filamentous bacteria. The most important group of biologically active compounds produced by microorganisms are antibiotics, of which about 100,000 tons are produced worldwide annually. Over half of these antibiotics are produced by actinomycetes, 10%–15% by non-filamentous bacteria and about 20% from filamentous fungi [2].

Macrosphelides (MS), are 16-membered, natural, macrolide polyketides first isolated in 1995 from a *Microsphaeropsis* sp. [3], which was derived from a soil sample [4]. So far, 13 natural macrosphelide isomers have been reported in the literature. They have been isolated from *Microsphaeropsis* sp. FO-5050 [3], *Periconia byssoides* OUPS-N133, the sea hare *Aplysia kurodai* [5,6], *Coniothyrium minitans* [7,8], the fungus, *Tritirachium* sp. HKI 0317 and dried sample of the Antarctic lichen *Neuropogon* sp. [9]. In addition, about twenty synthetic macrosphelides have been prepared [10]. Many molecules from this class of compounds showed promising biological activities. For example, macrosphelide H inhibited the adhesion of human leukemia HL-60 cells to human umbilical-vein endothelial cells (HUVEC) more potently than herbimycin A did [5]. The process of cell adhesion plays an important role in many pathological conditions, such as tumour development, allergies, and inflammatory diseases [11].

The most well-known of these compounds is macrosphelide A (MSA), which inhibited the mycelial growth of *Sclerotinia sclerotiorum* and *Sclerotium cepivorum* at a relatively low concentration of metabolite required to inhibit growth by 50% values (IG_50_, 46.6 and 2.9 μg/mL, respectively) [7]. This compound exhibited weak antimicrobial activity against *Bacillus subtilis*, *Staphylococcus aureus*, *Streptomyces viridochromogenes*, *Escherichia coli*, *Candida albicans* and *Aspergillus niger* [9]. Importantly, no acute toxicity was observed after an injection of MSA into BDF1 mice even at a dose of 200 mg/kg for five days [3].

In the present work, a strain of *Pleurotus ostreatus* was demonstrated to produce the macrosphelide (3*S*,9*S*,15*S*)-(6*E*,12*E*)-3,9,15-trimethyl-4,10,16-trioxacyclohexa-deca-6,12-diene-1,5,8,11,14-pentaone (**4**). This compound can induce apoptotic cell death in human lymphoma U937 cells (at 10 μM) and act as an effective sensitizer for hyperthermia-induced apoptosis [12]. To date, there have been no reports in the literature regarding *Pleurotus*-mediated biosynthesis of compounds from this group.

*Pleurotus ostreatus* (*Pleurotaceae*, higher *Basidiomycetes*), also known as the oyster mushroom or hiratake, is traditionally known as edible, delicious and one of the most cultivated mushrooms. This fungus is rich in protein, fiber, carbohydrates, vitamins, minerals and has a low calorie, fat and sodium content [13,14].

The white-rot fungus *Pleurotus ostreatus* has the ligninolytic system, which is composed of the enzymes laccase, aryl-alcohol oxidase, and two types of peroxidases: manganese-dependent peroxidase (MnP) and versatile peroxidase (VP). These enzymes can function separately and/or in cooperation [15]. These enzymes are also involved in the degradation of xenobiotic compounds, such as polycyclic aromatic hydrocarbons, industrial dyes and other soil pollutants, such as atrazine and carbamazepine [13,15].

The fungus has other known biological activities. For example, *P. ostreatus* mycelium extract, alone and in combination with cyclophosphamide, inhibited in vivo tumor growth in mice. The water extract of *P. ostreatus* exhibited the most significant cytotoxicity in comparison with many other mushroom extracts, by inducing the apoptosis of human carcinoma cells [16].

In addition, a lectin isolated from *P. ostreatus* not only potently inhibited the growth of sarcoma and hepatocellular carcinoma in mice, but also prolonged the lifespan of the mice. In turn, proteoglycans from *P. ostreatus* mycelia exerted immunomodulatory effects, including elevating mouse natural killer cell cytotoxicity and macrophage stimulation [16]. Different extracts of *P. ostreatus* had antimicrobial activity against *Bacillus subtilis*, *Escherichia coli*, *Pseudomonas aeruginosa*, *Salmonella typhi*, *Vibrio cholerae*, *Aspergillus niger* and *Fusarium oxysporum* [16].

In comparison with other microorganisms, little is known about the ability of *Pleurotus* genera to biotransform xenobiotic compounds. Car-3-ene was converted to many oxygenated derivatives by a dioxygenase of *P. sapidus* [17]. The same enzyme was responsible for the allylic oxidation of valencene to nootkatone and oxidation of the unsaturated fatty acid [18]. In a culture of *P. flabellatus* and *P. sajorcaju*, transformation of myrcene to myrcenol was observed [19]. In regard to steroids, 7-α-hydroxylation by cytochrome P450 monooxygenase of *Pleurotus* genus has been described [20].

Our previous studies dealt with the synthesis and biotransformation of terpenoid lactones. Filamentous fungi of genus *Fusarium* were mainly used for biotransformation until now. These fungi are able to carry out hydrolytic dehalogenation, thereby transforming halolactones into their corresponding hydroxylactones [21,22,23,24,25,26].

The aim of the study was to obtain a better knowledge of the biotransformation of selected halolactones. Due to the unexpected formation of macrosphelide, as a fungus secondary metabolite, several factors (salts or solvents) were checked as inductors of process.

## 2. Results and Discussion

Bromo- and iodolactone **1** and **2** with the *gem*-dimethylcyclohexane ring were selected as substrates for the biotransformations. The method of preparation was described in our earlier paper [23]. Strains from the Department of Chemistry’s collection, which were used in order to obtain new derivatives, had not been tested previously in the biotransformation of halolactones.

**Scheme 1:**
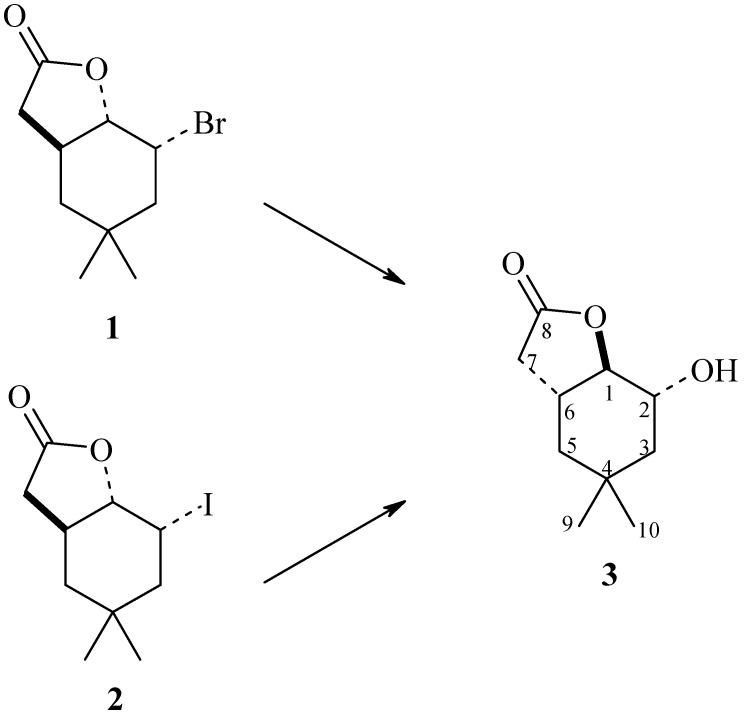
Biotransformatic hydrolytic dehalogenation of halolactones (**1**) and (**2**).

The following strains were used as bioreagents: *Yarrowia lipolytica* AM71, *Rhodotorula marina* AM77, *Rhodotorula rubra* AM82, *Penicillium vermiculatum* AM30, *Absidia glauca* AM254, *Absidia cylindrospora* AM336, *Penicillium frequentans* AM351, *Aspergillus ochraceus* AM456, and *Pleurotus ostreatus* AM482. It turned out that some of these microorganisms were able to perform a hydrolytic dehalogenation reaction, by a mechanism similar to S_N_2, producing hydroxylactone (**3**) (Scheme 1).

The results of the screening biotransformations are presented in Table 1.

As shown in Table 1, the selected yeast strains were not able to transform substrates **1** and **2** (entries 1–3). The same situation was observed in the case of *Aspergillus ochraceus* AM456 strain (entry 8). The next four strains transformed substrates **1** and **2** to the product **3**, to an extent not exceeding 50% (entries 4–7). Only the *Pleurotus ostreatus* AM482 strain was able to perform the complete conversion of both substrates, giving hydroxylactone **3** as the only product (entry 9, Scheme 1).

**Figure 1:**
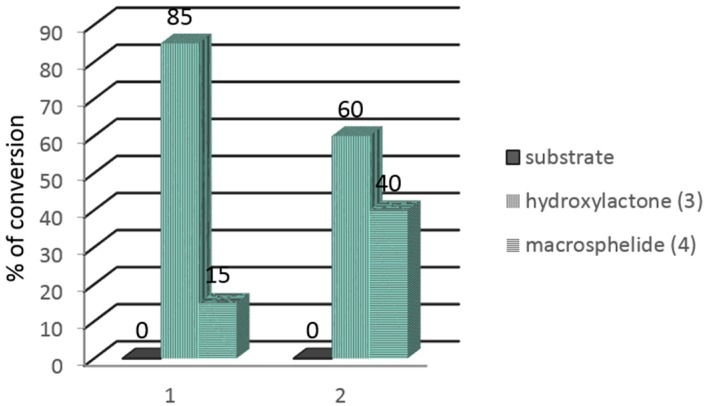
Results of the preparative biotransformation of bromo- and iodolactones **1** and **2** (in %) according to GC-FID.

In the next step, the *Pleurotus ostreatus* AM482 strain was used to carry out the preparative biotransformation of both substrates **1** and **2**. During the extraction of the preparative biotransformation products, chloroform was used instead of methylene chloride, which is commonly used by us for standard extractions [23,24,25]. In the GC chromatograms, in addition to the signals coming from the substrates **1**, **2** and the product **3**, a signal from another product **4** was present. The results of the preparative biotransformation are shown in Figure 1.

The preparative biotransformation carried out on bromolactone **1** gave 12.3 mg (16.5%) of hydroxylactone **3** and 7.1 mg of the additional product **4**. In turn, the use of iodolactone **2** as a substrate allowed for the production of 10.5 mg (16.8%) of hydroxylactone **3** and 12.8 mg of product **4**.

**Scheme 2:**
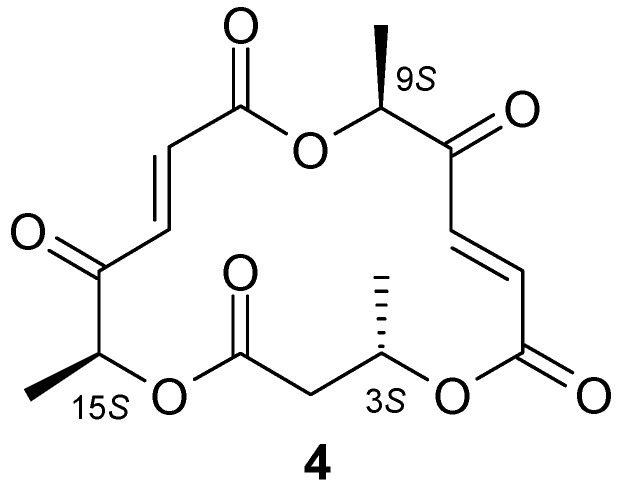
Structure of macrosphelide **4**.

^1^H-NMR analysis of hydroxylactone **3** confirmed that this compound was analogous to the hydroxylactone obtained in previous studies conducted by our team [23]. Analysis of the NMR spectra of the unexpected product **4** allowed us to determine that it was a metabolite of the fungus *Pleurotus ostreatus*. Compound **4** was identified as macrosphelide (6*E*,12*E*)-3,9,15-trimethyl-4,10,16-trioxacyclohexa-deca-6,12-diene-1,5,8,11,14-pentaone. It turned out that macrosphelide **4**, obtained during the biotransformation of halolactones **1** and **2**, was identical to the one described in an article by Sunazuka et al. [27]. Comparative analysis of the ^1^H-NMR and ^13^C-NMR spectra of the compound described in literature [28] with the biotransformation product obtained by us (Tables S1 and S2 in Supplementary Materials) confirmed that these were the same compound (Scheme 2).

The next step was to determine the optical rotation of product **4** obtained by the preparative biotransformation carried out on bromo- and iodolactone **1** and **2**. The obtained data were as follows: after the biotransformation of bromolactone **1** ${\left[\alpha \right]}_{D}^{20}$ = −57.27 (*c* = 0.42, CHCl_3_) and ${\left[\alpha \right]}_{D}^{20}$ = −61.79 (*c* = 0.62, CHCl_3_) after the biotransformation of iodolactone **2**.

The chemical synthesis of the macrosphelide, and the absolute configurations of three chiral centres present in the molecule, was presented in the paper mentioned earlier [27]. Therefore, a collaboration with Yuji Matsuya and Kenji Sugimoto was established, who also received this macrosphelide [28]. Their compound was characterized by an optical rotation of ${\left[\alpha \right]}_{D}^{19}$ = −58 (*c* = 0.24, CHCl_3_). We achieved very similar results: after the biotransformation of bromolactone **1** ${\left[\alpha \right]}_{D}^{20}$ = −54.5 (*c* = 0.25, CHCl_3_) and ${\left[\alpha \right]}_{D}^{20}$ = −57 (*c* = 0.24, CHCl_3_) after biotransformation of iodolactone **2**.

The results of the rotation determination of the synthesised compound and macrosphelide obtained by biotransformation proved to be very close to the same value, and identical to the mark. On this basis, we assumed that macrosphelide **4** isolated from oyster mushroom has the absolute configuration 3*S*, 9*S*, 15*S*. It is worth noticing that compound **4** was previously obtained exclusively by chemical synthesis and so far there is no information in the literature about the possibility of its biosynthesis by fungi or yeast.

Because we used chloroform instead of methylene chloride for the extraction of the preparative biotransformation, we discovered that the *Pleurotus ostreatus* AM482 strain was able to produce macrosphelide **4**, a compound obtained only by organic synthesis previously. In order to determine the effect of the extraction solvent, the experiment was repeated, this time extracting the product with a portion of methylene chloride and a second portion with chloroform. This experiment revealed that the methylene chloride extraction showed only the signal derived from the hydroxylactone **3** in the GC-FID chromatogram. When chloroform was used, two signals were observed in the GC-FID chromatograms: the first coming from the hydroxylactone **3** and the second from the macrosphelide **4**. The control experiment (without added substrate) conducted in parallel demonstrated that macrosphelide **4** was produced only in the presence of iodolactone **2**.

Continuing our research we decided to find out the effect of temperature on the behaviour of the *Pleurotus ostreatus* strain. It was found that the optimum temperature for growth and biotransformation were in the range of 25 °C to 26 °C. At the lower temperature (21 °C–22 °C) the increase in biomass was very slow, so the substrate did not undergo any transformation. In turn, higher temperatures (28 °C–30 °C) resulted in dieback of the culture, and thereby inhibition of the biotransformation. The optimum temperature for the growth of the culture in the present work was consistent with the literature data [29].

Iodolactone **2** was transformed into hydroxylactone **3**, and simultaneously induced the formation of macrosphelide **4**, providing that the biotransformation was carried out at a temperature of 25 °C–26 °C. Lowering or raising the temperature by a few degrees dramatically reduced the substrate conversion and thus none of these products were formed.

As shown in our previous studies [21,22,23,24,25,26], conversion of iodolactone to hydroxylactone was followed by hydrolytic dehalogenation of the halolactone. Consequently, in the medium where biotransformation takes place, iodide ions may be found along with iodolactone.

In another experiment, the effect of substrate (iodolactone **2**) addition and a potential inducer (KI) was tested. This time, both compounds (10 mg) were added to the cultures, which were either in the log growth phase (three days) or in the stationary phase (six days). The extractions were carried out three, five, seven and nine days after the addition of substrate. The results are presented in Table 2 and Table 3.

Cultures to which the substrate was added after three days showed the presence of macrosphelide **4** after an additional three days. Interestingly, this compound was also found in the cultures to which potassium iodide was added, which suggested that the production of macrosphelide **4** was induced by iodolactone **2**. It is worth noting that *P. ostreatus* is one of the few fungal species resistant to potassium iodide that maintains the ability to accumulate iodine [30]. However, there are no literature data about the effects of iodine on the metabolism of *Pleurotus*.

When substrate (**2**) or KI was added to six day cultures in both cases macrosphelide was also present, but in a smaller amount. After nine days of biotransformation, macrosphelide **4** completely disappeared, probably due to further metabolism of this compound by the microorganism.

Another experiment was designed to test whether macrosphelide **4** was produced only under the influence of halolactone and potassium iodide, or also under the influence of other compounds that might induce secondary metabolites in fungal cultures. The putative inducers were added to flasks containing 100 mL of three day old cultures of *Pleurotus ostreatus* AM482 strain. The results of this phase of the study are presented in Table 4.

Because halolactones **1** and **2** and potassium iodide induced the synthesis of macrosphelide **4**, we evaluated whether or not potassium bromide or sodium chloride could also induce the production of this molecule. Neither of these salts was effective. It is known from the literature [32] that stress conditions can promote the production of antibiotics; it is also known that *Pleurotus ostreatus* is sensitive to salinity [29]. Because of these facts, NaCl was added to the culture (final concentration of 0.1 M) to induce osmotic stress. It appeared that this strain behaved differently, since a high concentration of salt increased the amount of biomass without inducing the production of macrosphelide **4**. Organic compounds such as ethanol, dimethylsulphoxide (DMSO), and hydrogen peroxide have been used previously to elicit secondary metabolite biosynthesis [31]. These data encouraged us to use these compounds as inducers of macrosphelide **4** production, but these experiments have proven unsuccessful.

The Tomprefa group [8] postulated that macrosphelides were synthesized by the polyketide pathway. Since it is known that the biosynthesis of other macrolide antibiotics, erythromycin for example, are stimulated by propionic acid and propanol [33], we decided to use these compounds in our research. Unfortunately, they also proved to be ineffective. Propanol was toxic to microorganisms in concentration similar to ethanol. When propanol was added in a lower concentration, only traces of compound **4** were observed. It is possible that macrosphelide **4** is synthesized by the *Pleurotus ostreatus* strain by another route or that the strain does not have the enzyme linking alcohol and propionic acid with coenzyme A.

In a review on the effect of heavy metals on secondary metabolite production/cellular differentiation, Weinberg [34] summarizes evidence that regulation primarily occurs at the level of transcription. Furthermore, three heavy metal ions, manganese, ferric and zinc, were identified as having a key role in the secondary metabolism of a wide variety of microorganisms [31]. In addition, *Pleurotus ostreatus* has the ability to accumulate metals such as iron, zinc, manganese, copper, and nickel [35,36].

In our case, the presence of iron, copper, manganese and cobalt induced the biosynthesis of the macrosphelide. Surprisingly, of the selected metals, only zinc ions proved to be completely inactive, even though it is known from the literature that in *Aspergillus parasiticus*, zinc overload suppressed the formation of the polyketide, versicolorin A [34]. Such an effect may be due to the fact that zinc is the only one of these metal ions which is not involved in redox processes. Alternatively, the ineffectiveness of zinc could be caused by problems with the transport of this metal into the cell and thus its bioavailability.

The best effect brought the addition of EDTA chelated iron salts, wherein the degree of oxidation of the iron influenced to a small extent the production of macrosphelide **4**. It is known from the literature [37] that *Pleurotus* actively transports iron into the cell by redox processes. It is worth noting that iron plays many functions in cells, it is a component of haem among others. Haem is a cofactor for many enzymes, including oxygenase, which is involved in the biosynthesis of polyketide antibiotics. Enzymes participating in the biosynthesis of the polyketide antibiotic amphotericin by *Streptomyces nodosus* cultures contain a cysteine residue that coordinates with the haem iron [38].

Because both iron and manganese ions induced the formation of macrosphelide **4**, it is feasible that the limiting step in the biosynthesis of this compound are the reactions catalysed by enzymes associated with cytochrome P450; P450 monooxygenase or manganese-dependent peroxidase. Further research on the biosynthesis of macrosphelide **4** is in progress.

## 3. Experimental Procedures

### 3.1. General Information

The progress of biotransformation and the purity of products were checked on silica gel-coated aluminum plates (DC-Alufolien Kieselgel 60 F254, Merck: Darmstadt, Germany ) and by GC analysis carried out on a 6890N instrument (Varian, Agilent Technologies, Santa Clara, CA, USA) using an DB-17 column (cross-linked methyl silicone gum, 30 m × 0.32 mm × 0.25 μm). The temperatures during the GC analysis were as follows: injector 150 °C, detector (FID) 280 °C, column temperature: 120 °C, 120 °C–280 °C (rate 25 °C/min), 280 °C (hold 1 min). Products of biotransformation were purified using preparative column chromatography on silica gel (Kieselgel 60, 230–400 mesh). Optical rotation was measured in chloroform solutions on a P-2000 polarimeter (Jasco Easton, PA, USA). NMR spectra were recorded in a CDCl_3_ solution on an Avance™ 600 MHz spectrometer (Bruker, Billerica, MA, USA) or a UNITYplus 500 (500 MHz) spectrometer and a Gemini 300 (75 MHz) (Varian, Palo Alto, CA, USA).

### 3.2. Biotransformation

#### 3.2.1. Microorganisms

The yeast and fungal strains were obtained from a collection of the Institute of Biology and Botany, Medical University, Wroclaw, Poland: *Yarrowia lipolytica* AM71, *Rhodotorula marina* AM77, *Rhodotorula rubra* AM82, *Penicillium vermiculatum* AM30, *Absidia glauca* AM254, *Absidia cylindrospora* AM336, *Penicillium frequentans* AM351, *Aspergillus ochraceus* AM456, *Pleurotus ostreatus* AM482. All these strains are available in the Department of Chemistry, Wroclaw University of Environmental and Life Sciences. The strains were cultivated on Sabouraud’s agar containing 0.5% of aminobac, 0.5% of peptone, 4% of glucose and 1.5% of agar dissolved in distilled water at 28 °C and stored in a refrigerator at 4 °C.

#### 3.2.2. Screening Procedure of Substrates Biotransformation

The yeast and fungal strains which were used for biotransformation were cultivated at 25 °C in 300 mL Erlenmayer flasks, containing 100 mL of the medium consisting of glucose (3 g) and peptobac (1 g). After three days, 10 mg of the substrate **1** or **2** dissolved in 1 mL of acetone was added to each flask. The shaken cultures were incubated with the substrate for nine days. After three, five, seven and nine days of incubation, the mixture of unreacted substrate, products and mycelium was extracted with 15 mL of dichloromethane or chloroform. These solutions were dried over anhydrous magnesium sulphate and then filtered. After evaporation of the solvent, the residue was dissolved in 2 mL of acetone and analyzed by TLC (hexane–acetone, 3:1) and GC (DB-17 column).

#### 3.2.3. Preparative Biotransformations

Ten Erlenmeyer flasks with the three-day cultures of *Pleurotus ostreatus* were prepared as described in the screening procedure. Then 100 mg of the halolactones **1** or **2** were dissolved in 10 mL of acetone and distributed between these flasks. After nine days the products were extracted with chloroform (3 × 40 mL). The combined organic solutions were dried over anhydrous magnesium sulphate and the solvent was evaporated in vacuo. The mixture containing the products, the unreacted substrate and the fungal metabolites was separated by column chromatography (silica gel, hexane:acetone, 3:1) to obtain the pure products—hydroxylactone **3** and macrocyclic lactone **4**.

*2-Hydroxy-4,4-dimethyl-9-oxabicyclo[4.3.0]nonan-8-one* (**3**): ^1^H-NMR (600 MHz, CDCl_3_) δ: 0.95 (s, 3H, CH_3_-9), 1.00 (s, 3H, CH_3_-10), 1.03 (m, 1H, one of CH_2_-5), 1.38 (dd, *J* = 12.6 and 12.5 Hz, 1H, one of CH_2_-3), 1.43 (dd, *J* = 13.9 and 6.2 Hz, 1H, one of CH_2_-5), 1.66 (m, 1H, one of CH_2_-3), 1.99 (s, 1H, OH), 2.27 (d, *J* = 16.7 Hz, 1H, one of CH_2_-7), 2.60 (dddd, *J* = 12.3, 6.4, 6.2 and 3.8 Hz, 1H, H-6), 2.77 (dd, *J* = 16.7 and 6.4 Hz, 1H, one of CH_2_-7), 3.95 (m, 1H, H-2), 4.58 (t, *J* = 3.8 Hz, 1H, H-1).

*(3S,9S,15S)-(6E,12E)-3,9,15-Trimethyl-4,10,16-trioxacyclohexa-deca-6,12-diene-1,5,8,11,14-pentaone* (**4**): ^1^H-NMR (600 MHz, CDCl_3_) δ: 1.44 (d, *J* = 6.3 Hz, 3H, CH_3_-C-3), 1.49 (d, *J* = 7.0 Hz, 3H, CH_3_-C-15), 1.61 (d, *J* = 7.2 Hz, 3H, CH_3_-C-9), 2.70 (dd, *J* = 16.6 and 2.0 Hz, 1H, one of CH_2_-2), 2.92 (dd, *J* = 16.6 and 11.3 Hz, 1H, one of CH_2_-2), 5.22 (q, *J* =6.9 Hz, 1H, H-15), 5.29 (q, *J* =7.1 Hz, 1H, H-9), 5.40 (m, 1H, H-3), 6.66 (d, *J* = 16.0 Hz, 1H, H-6), 6.87 (d, *J* = 15.8 Hz, 1H, H-13), 7.18 (d, *J* = 15.8 Hz, 1H, H-12), 7.38 (d, *J* = 16.0 Hz, 1H, H-7), ^13^C-NMR (151 Hz, CDCl_3_), δ: 15.93 (CH_3_C-15), 17.07 (CH_3_C-9), 19.52 (CH_3_C-3), 40.64 (C-2), 69.22 (C-3), 75.56 (C-15), 76.35 (C-9), 132.03 (C-6), 132.30 (C-13), 132.33 (C-12), 134.38 (C-7), 163.11 (C-1), 163.49 (C-8), 170.11 (C-14), 195.61 (C-5), 197.46 (C-11).

#### 3.2.4. The Influence of Various Factors on the Production of Macrosphelide **4**

##### Temperature

Six Erlenmeyer flasks with the three-day cultures of *P. ostreatus* were prepared as described in the screening procedure. The culture was grown at three different temperatures: 21 °C–22 °C, 25 °C–26 °C and 28 °C–30 °C. After three, five, seven and nine days of biotransformation, one-fourth the volume of the medium with overgrown mycelium was taken from each flask. Such samples were extracted with chloroform, dried and analysed by means of GC.

##### Growth Phase of the Microorganism

Substrate **2** was added to a culture of *P. ostreatus* after three or six days. After three, five, seven and nine days of biotransformation the culture was extracted with chloroform and analysed by means of GC. As a control, in one flask the microorganism was grown without substrate **2**.

##### Induction of Macrosphelide (**4**) Production

Eighteen Erlenmeyer flasks with three-day cultures of *P. ostreatus* were prepared as described in the screening procedure. To each flask was added, respectively KI (5 mg), KBr (5 mg), NaCl (3 mg), NaCl (585 mg), DMSO (1 mL), H_2_O_2_, (2 mL, 3%), acetone (1 mL), ethanol (1.2 mL), propanol (0.75 mL), propionic acid (0.38 mL), FeSO_4_ × 7H_2_O (5 mg) and EDTA (25 mg), FeCl_3_ × 6H_2_O (5 mg) and EDTA (25 mg), CuSO_4_ × 5H_2_O (5 mg) and EDTA (25 mg), CuCl (5 mg) and EDTA (25 mg), CoCl_2_ × 6H_2_O (5 mg) and EDTA (25 mg), ZnCl_2_ (5 mg) and EDTA (25 mg), MnCl_2_ (5 mg) and EDTA (25 mg). The salts were dissolved in deionized water. In addition, a control experiment, without any salt, was also performed. After seven days the cultures were extracted with chloroform and analysed by means of GC.

## 4. Conclusions

From the nine tested microorganism strains, four were able to biotransform bromolactone **1**, whereas iodolactone **2** was transformed by five strains. A complete conversion of the substrate was observed only in the *Pleurotus ostreatus* culture. Both substrates were converted into hydroxylactone **3** by hydrolytic dehalogenation. When chloroform was used for the extraction instead of methylene chloride, the presence of a fungal metabolite, macrosphelide **4**, was observed in the extract in addition to hydroxylactone **3**. Further studies have demonstrated that the biosynthesis of macrosphelide **4** was induced not only by substrates **1** and **2**, but also by iodide, bromide, iron, copper, manganese and cobalt ions. The present work is the first to discover the production of macrosphelide **4** in *Pleurotus ostreatus* culture and to investigate the induction of its biosynthesis.

## Figures and Tables

**Table 1:** Results of the screening biotransformation of bromo- and iodolactones **1** and **2** after nine days (according to GC-FID).

No.	Strain	Substrate (1) (%)	Product (3) (%)	Substrate (2) (%)	Product (3) (%)
1	*Y. lipolytica* AM71	100	n.d.	100	n.d.
2	*R. marina* AM77	100	n.d.	100	n.d.
3	*R. rubra* AM82	100	n.d.	100	n.d.
4	*P. vermiculatum* AM30	58.8	41.2	69.0	31.0
5	*A. glauca* AM254	54.4	45.6	57.7	42.3
6	*A. cylindrospora* AM336	100	n.d.	73.9	26.1
7	*P. frequentans* AM351	57.8	42.2	64.7	35.3
8	*A. ochraceus* AM456	100	n.d.	100	n.d.
9	*P. ostreatus* AM482	n.d.	**100**	n.d.	**100**

n.d.—not detected.

**Table 2:** The results obtained with iodolactone **2** in the log growth phase (according to GC-FID).

Time of Biotransformation (Days)	Iodolactone (2) (%)	Hydroxylactone (3) (%)	Macrosphelide (4) (%)
3	55.7	10.3	34.0
5	30.2	17.9	51.9
7	17.2	25.5	57.3
9	n.d.	65.0	35.0

n.d.—not detected.

**Table 3:** The results obtained with iodolactone **2** addition in stationary phase of growth (according to GC-FID).

Time of Biotransformation (Days)	Iodolactone (2) (%)	Hydroxylactone (3) (%)	Macrosphelide (4) (%)
3	56.7	n.d.	43.3
5	11.6	45.4	43.0
7	n.d.	51.9	48.1
9	n.d.	100	n.d.

n.d.—not detected.

**Table 4:** Potential impact of inducers on macrosphelide **4** production after 7 days (according to GC-FID).

No	Inducer	Amount	Result *
1	Bromolactone **1**	10 mg	+
2	Iodolactone **2**	10 mg	+++
3	Acetone	1 mL	-
4	DMSO	1 mL **	-
5	H_2_O_2_	2 mL **	-
6	Ethanol	1.2 mL **	-
7	Propanol	0.75 mL **	+
8	Propionic acid	0.38 mL **	-
9	NaCl	3 mg–585 mg	-
10	KBr	5 mg	+
11	KI	5 mg	+++
12	Fe^2+^	5 mg	+++
13	Fe^3+^	5 mg	++
14	Cu^2+^	5 mg	+++
15	Cu^+^	5 mg	+
16	Co^2+^	5 mg	++
17	Zn^2+^	5 mg	-
18	Mn^2+^	5 mg	++

* (-) no detectable production of **4**; (+) trace production (0.02–0.05 mg/mL); (++) medium production (0.05–0.10 mg/mL); (+++) high production (0.10–0.15 mg/mL); ** values according to the cited literature [31].

## Supplementary Materials

Supplementary materials can be accessed at: http://www.mdpi.com/1420-3049/21/7/859/s1.
