# Supplementary material for: A Macrosphelide as the Unexpected Product of a Pleurotus ostreatus Strain-Mediated Biotransformation of Halolactones Containing the gem-Dimethylcyclohexane Ring. Part 1
Source: Molecules. 2016 Jun 30;21(7):859. doi: 10.3390/molecules21070859 (PMC6273929; doi:10.3390/molecules21070859)
Supplement: Supplementary file 1 [file molecules-21-00859-s001.pdf]

Supplementary Materials: A Macrosphelide as the Unexpected Product of a *Pleurotus ostreatus* Strain-Mediated Biotransformation of Halolactones Containing the *gem*-dimethylcyclohexane Ring. Part 1

Katarzyna Wińska, Wanda Mączka, Małgorzata Grabarczyk, Kenji Sugimoto, Yuji Matsuya, Antoni Szumny and Mirosław Anioł

Table S1. Comparison of ^{1}H-NMR data (600 MHz for microbial and 500 MHz for synthetic macrosphelide, in $CDCl_3$ in both cases).

Proton	Microbial Macrosphelide 4	Synthetic Macrosphelide 4
CH_3-C-3	1.44 (d, *J* = 6.3 Hz)	1.42 (d, *J* = 5.5 Hz)
CH_3-C-15	1.49 (d, *J* = 7.0 Hz)	1.47 (d, *J* = 7.5 Hz)
CH_3-C-9	1.61 (d, *J* = 7.2 Hz)	1.58 (d, *J* = 7.0 Hz)
CH_2-2	2.70 (dd, *J* = 16.6 and 2.0 Hz)	2.70 (dd *J* = 16.5 and 2.5 Hz)
CH_2-2	2.92 (dd, *J* = 16.6 and 11.3 Hz)	2.90 (dd, *J* = 16.5 and 11.5 Hz)
H-15	5.22 (q, *J* = 6.9 Hz)	5.20 (q, *J* = 7.0 Hz)
H-9	5.29 (q, *J* = 7.1 Hz)	5.28 (q, *J* = 7.0 Hz)
H-3	5.40 (m)	5.38 (m)
H-6	6.66 (d, *J* = 16.0 Hz)	6.64 (d, *J* = 16.0 Hz)
H-13	6.87 (d, *J* = 15.8 Hz)	6.87 (d, *J* = 16.0 Hz)
H-12	7.18 (d, *J* = 15.8 Hz)	7.16 (d, *J* = 16.0 Hz)
H-7	7.38 (d, *J* = 16.0 Hz)	7.36 (d, *J* = 16.0 Hz)

Table S2. Comparison of ^{13}C-NMR data (600 MHz for microbial and 500 MHz for synthetic macrosphelide, in CDCl3 in both cases).

Carbon	Microbial Macrosphelide 4	Synthetic Macrosphelide 4
CH_3C-15	15.93	15.91
CH_3C-9	17.07	17.04
CH_3C-3	19.52	19.48
C-2	40.64	40.59
C-3	69.22	69.19
C-15	75.56	75.53
C-9	76.35	76.32
C-6	132.03	132.01
C-13	132.30	132.27
C-12	132.33	132.30
C-7	134.38	134.34
C-1	163.11	163.07
C-8	163.49	163.47
C-14	170.11	170.09
C-5	195.61	195.59
C-11	197.46	197.44

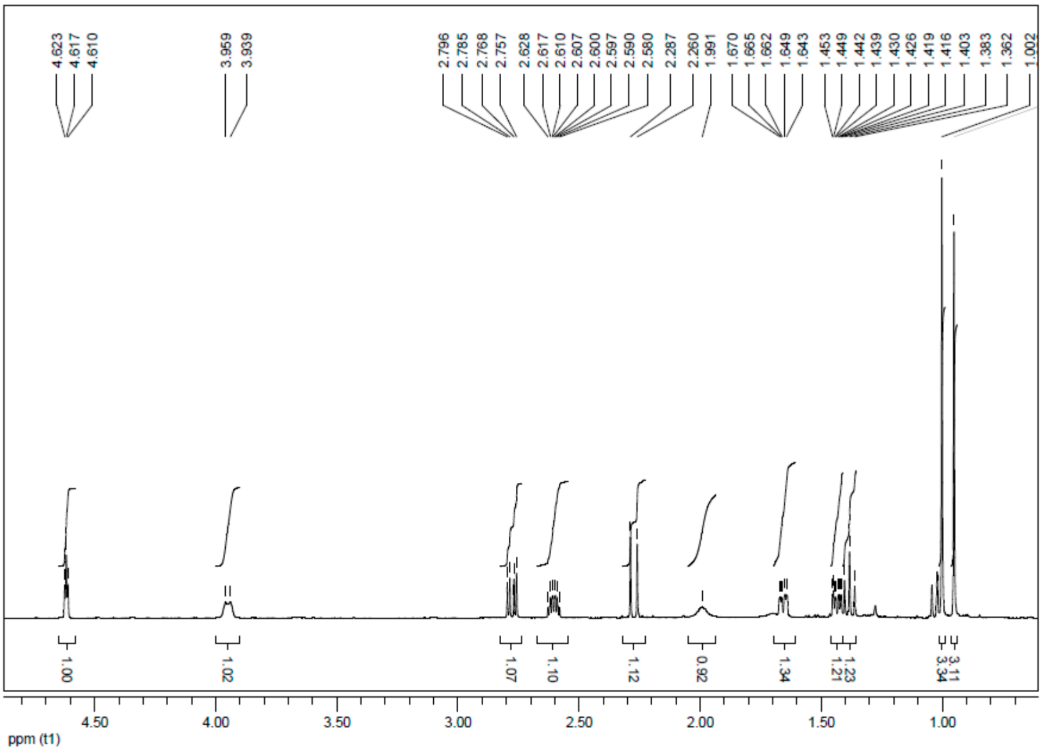

Figure S3. ^{1}H-NMR (600 MHz) spectrum of hydroxylactone **3** in $CDCl_3$.

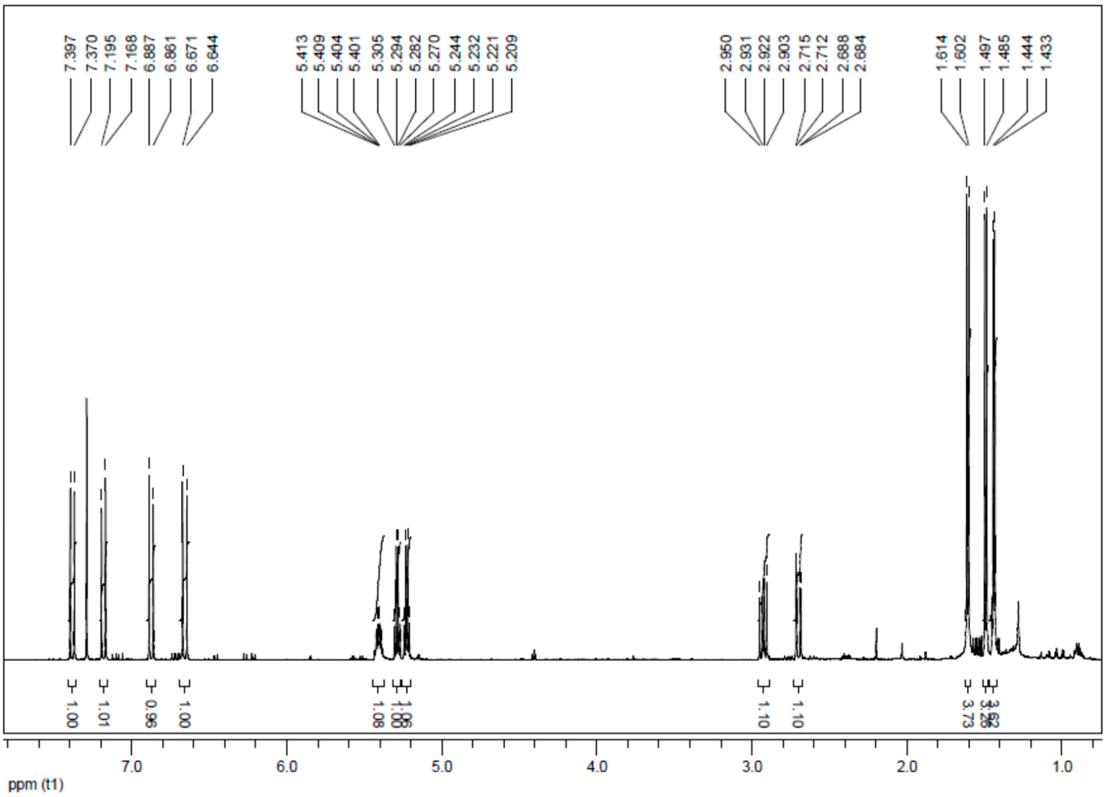

Figure S4. ^{1}H-NMR (600 MHz) spectrum of microbiologically obtained macrosphelide **4** in $CDCl_3$.

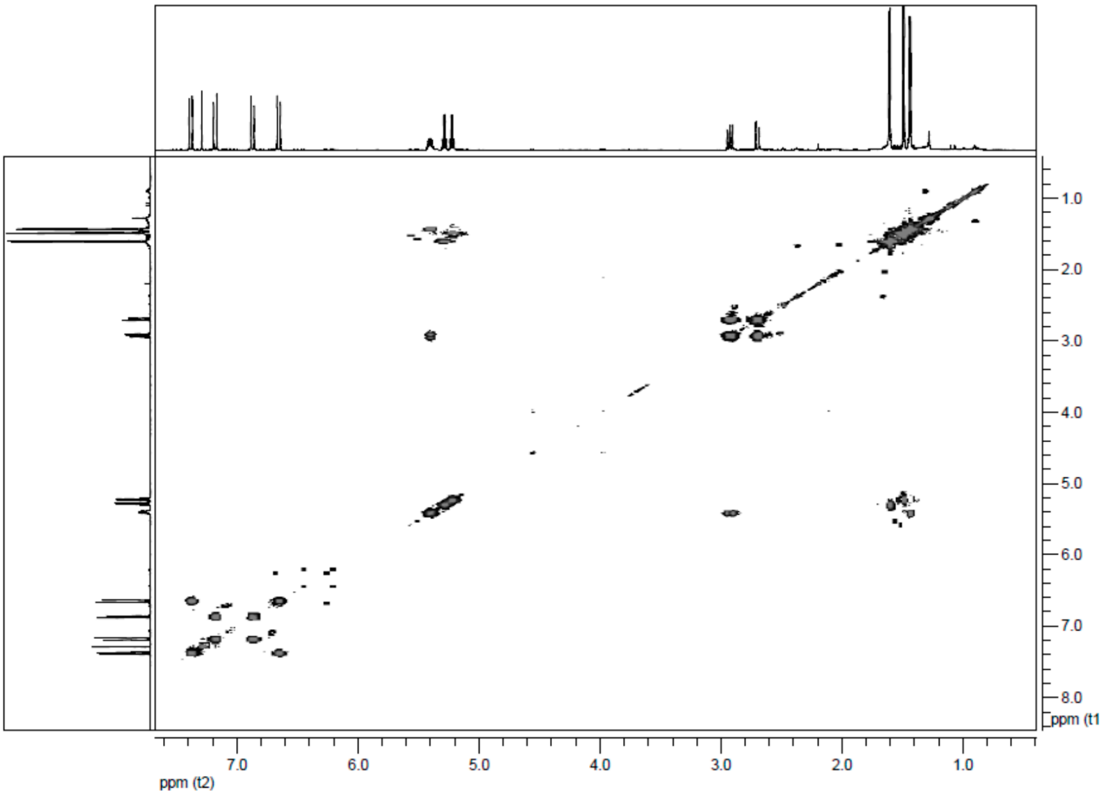

Figure S5. COSY NMR (151 MHz) spectrum of microbiologically obtained macrosphelide **4** in $CDCl_3$.

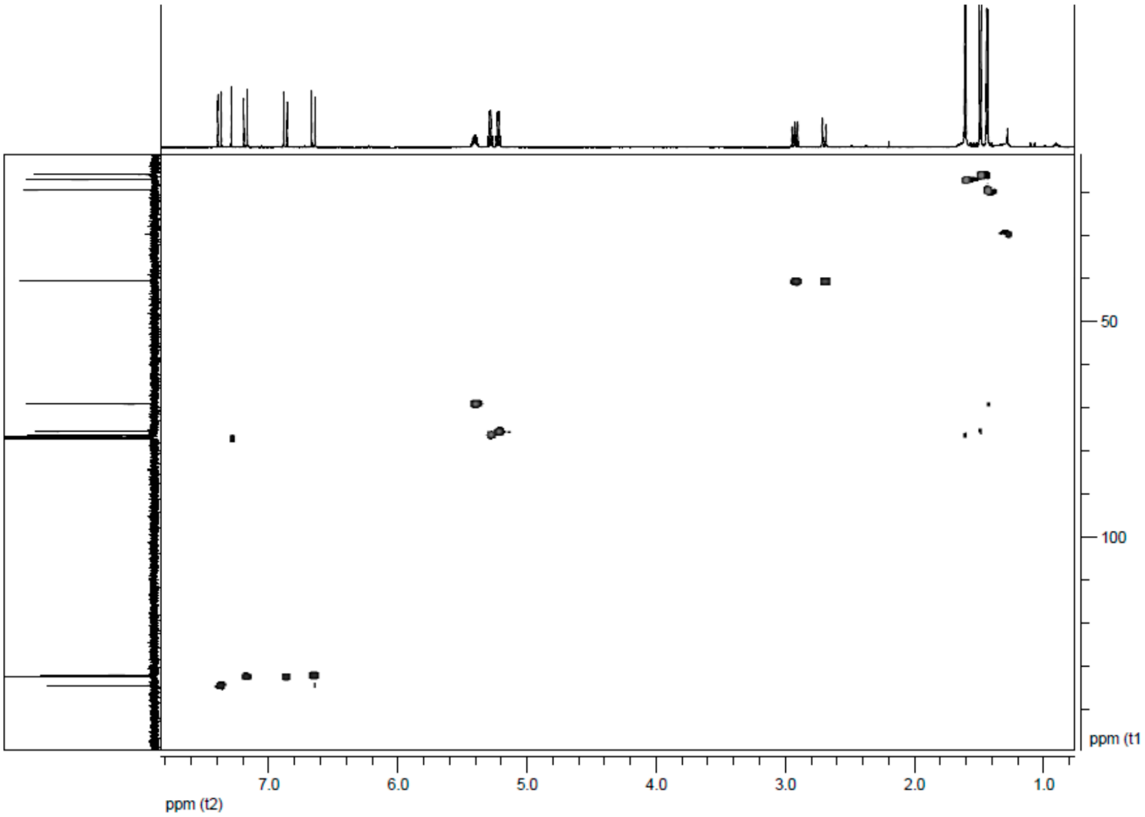

Figure S6. HMQC NMR (151 MHz) spectrum of microbiologically obtained macrosphelide **4** in $CDCl_3$.

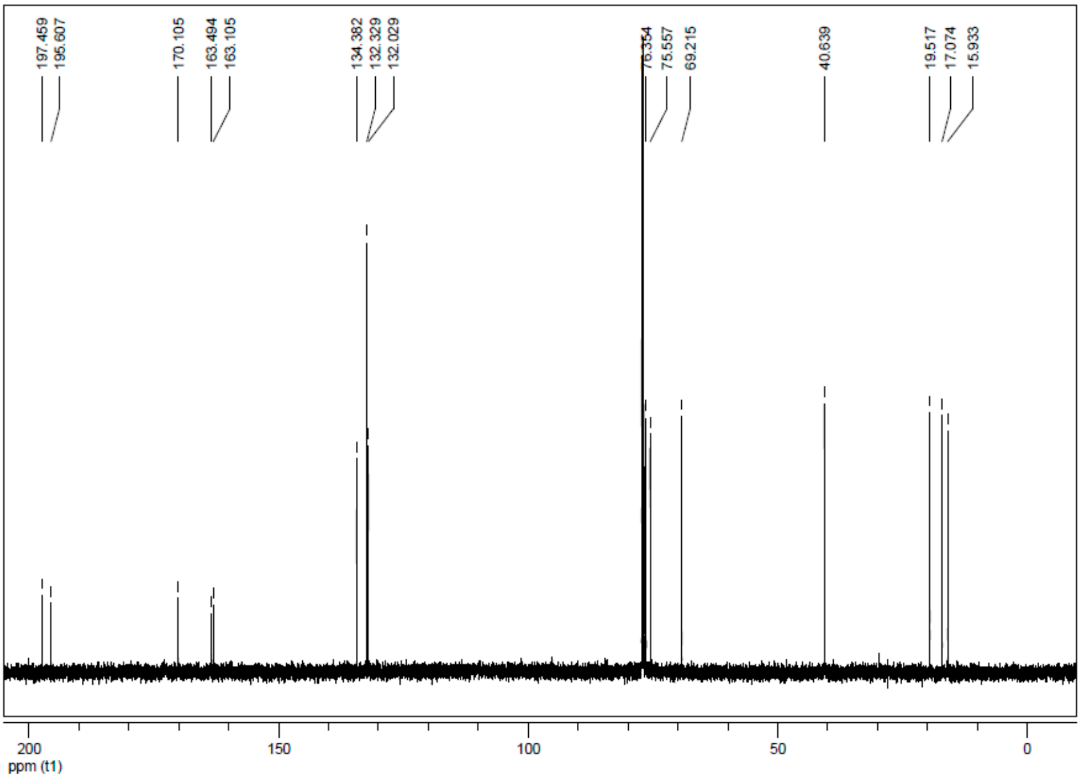

Figure S7. ^{13}C-NMR (151 MHz) spectrum of microbiologically obtained macrosphelide **4** in $CDCl_3$.

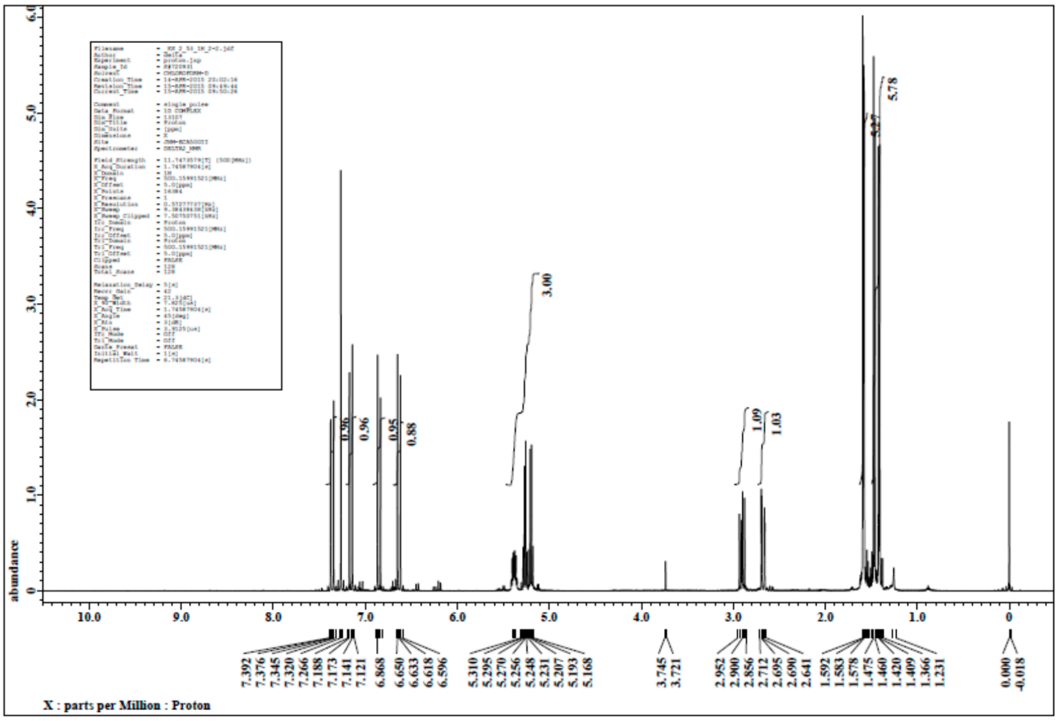

Figure S8. ^{1}H-NMR (500 MHz) spectrum of synthetically obtained macrosphelide **4** in $CDCl_3$.

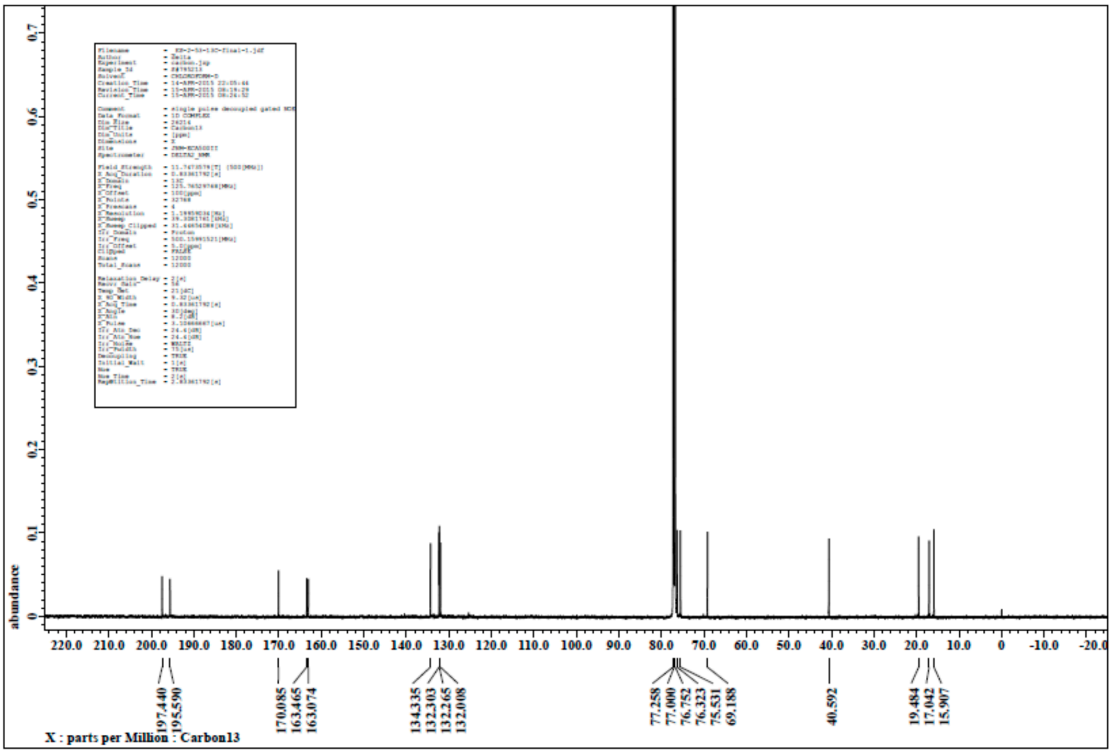

Figure S9. ^{13}C-NMR (75 MHz) spectrum of synthetically obtained macrosphelide **4** in $CDCl_3$.

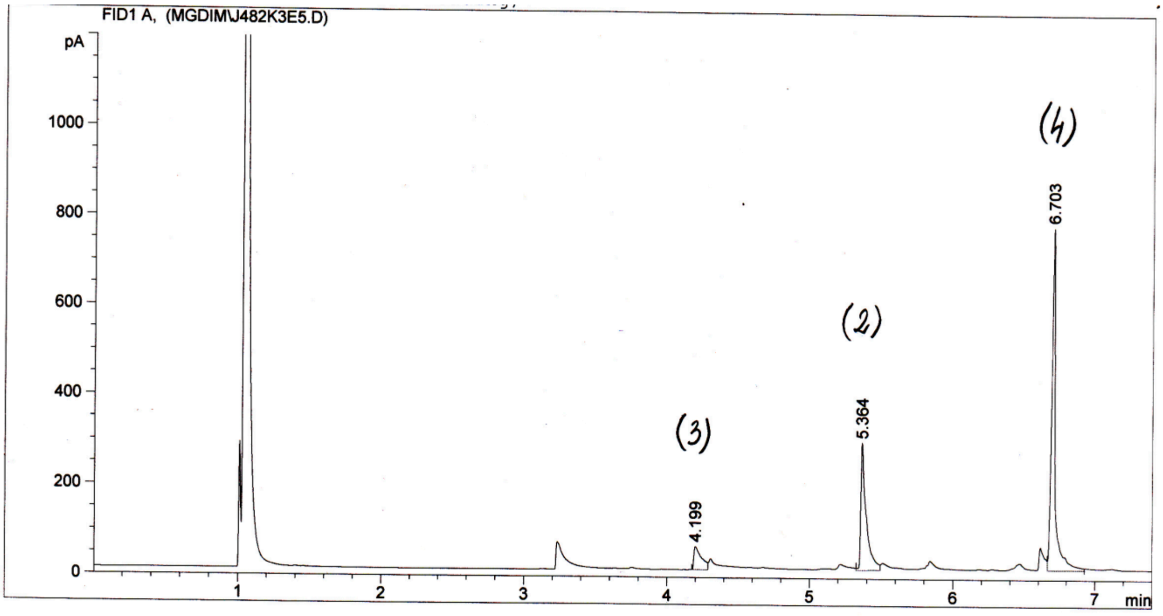

Figure S10. GC-chromatogram of chloroform extract obtained after biotransformation of iodolactone **2** (**2**-substrate, **3**-hydroxylactone, **4**-macrosphelide).

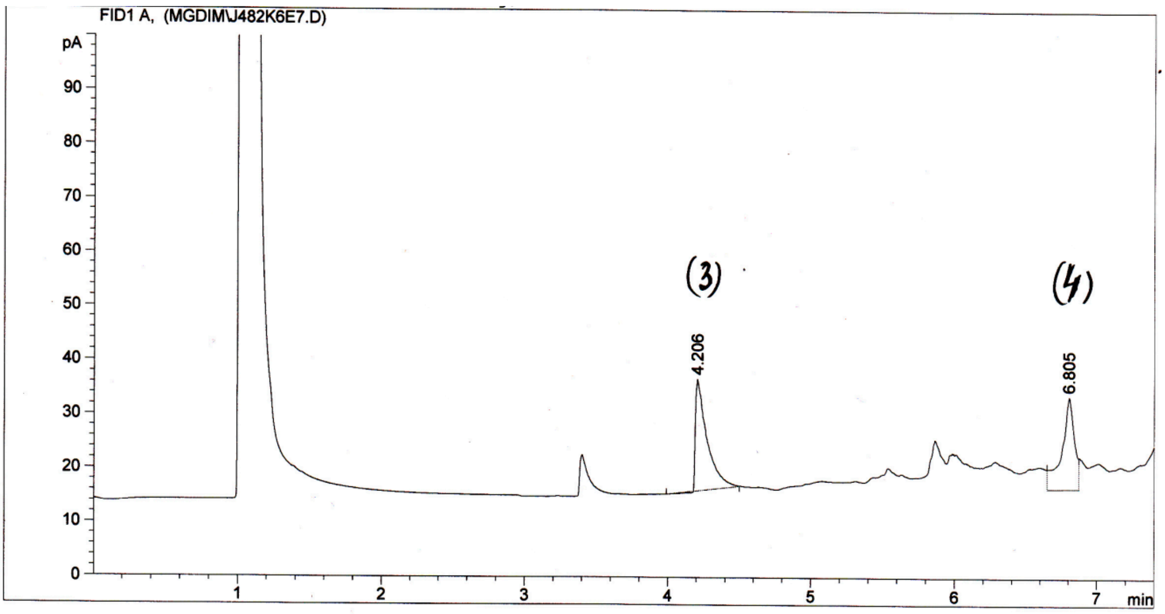

Figure S11. GC-chromatogram of chloroform extract obtained after biotransformation of iodolactone **2** (**3**-hydroxylactone, **4**-macrosphelide).

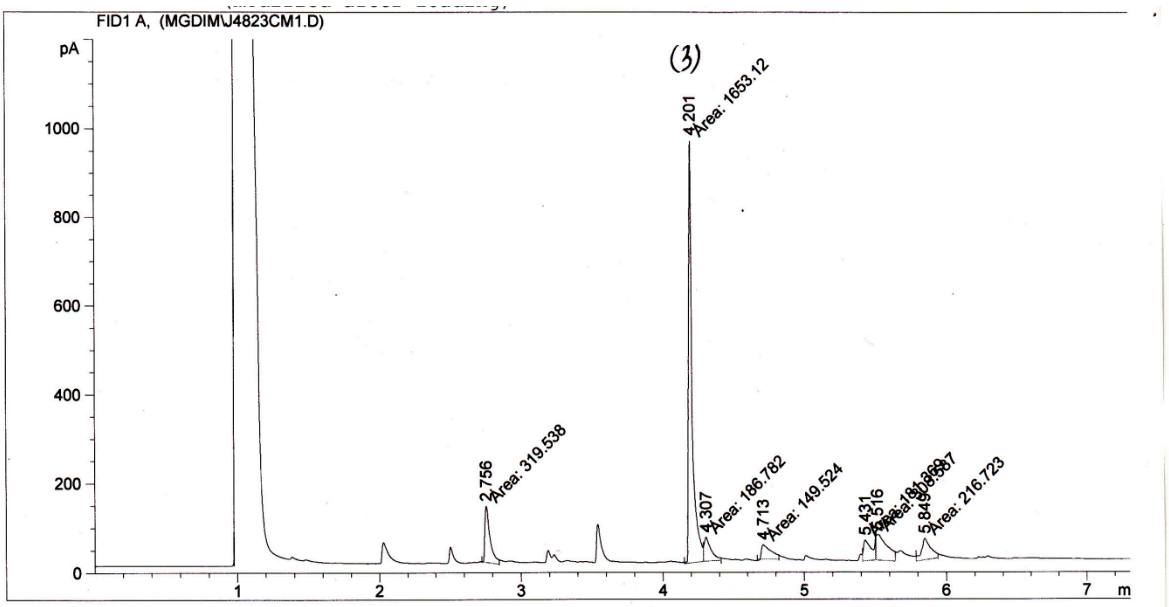

Figure S12. GC-chromatogram of methylene chloride extract after biotransformation of iodolactone **2** (**3**-hydroxylactone).
